# Football Fan Aggression: The Importance of Low Basal Cortisol and a Fair Referee

**DOI:** 10.1371/journal.pone.0120103

**Published:** 2015-04-06

**Authors:** Leander van der Meij, Fabian Klauke, Hannah L. Moore, Yannick S. Ludwig, Mercedes Almela, Paul A. M. van Lange

**Affiliations:** 1 Department of Social and Organizational Psychology, VU University Amsterdam, Amsterdam, The Netherlands; 2 Laboratory of Social Cognitive Neuroscience, Department of Psychobiology, Valencia, University of Valencia, Valencia, Spain; Radboud University Nijmegen, NETHERLANDS

## Abstract

Fan aggression in football (soccer) is a societal problem that affects many countries worldwide. However, to date, most studies use an epidemiological or survey approach to explain football fan aggression. This study used a controlled laboratory study to advance a model of predictors for fan aggression. To do so, football fans (*n* = 74) saw a match summary in which their favorite team lost against their most important rival. Next, we measured levels of aggression with the hot sauce paradigm, in which fans were given the opportunity to administer a sample of hot sauce that a rival football supporter had to consume. To investigate if media exposure had the ability to reduce aggression, before the match fans saw a video in which fans of the rival team commented in a neutral, negative, or positive manner on their favorite team. Results showed that the media exposure did not affect aggression. However, participants displayed high levels of aggression and anger after having watched the match. Also, aggression was higher in fans with lower basal cortisol levels, which suggests that part of the aggression displayed was proactive and related to anti-social behavior. Furthermore, aggression was higher when the referee was blamed and aggression was lower when the performance of the participants’ favorite team was blamed for the match result. These results indicate that aggression increased when the match result was perceived as unfair. Interventions that aim to reduce football fan aggression should give special attention to the perceived fairness of the match result.

## Introduction

Football fan aggression is a societal problem that affects many countries worldwide (referring to association football, i.e., soccer, and not American football). For example, fan aggression frequently results in injuries and extensive property destruction and even has resulted in deaths [1]. It appears that those spectators that mostly engage in violence are fanatic fans; for example, self-proclaimed fanatic Italian fans indicate they participate more in these disturbances than moderate Italian fans (*n* = 505) [2]. The socio-demographic profile of these fanatic fans is that they are mostly young men with blue collar jobs. To illustrate, in a sample of 246 fanatic Italian football fans (Ultras), 83% were men, only 3% were older than 30, and 80% had blue collar jobs [3]. The research reported in this paper seeks to disentangle some of the contextual factors that have the potential to increase football fan aggression. To do so, we performed a controlled laboratory study, which design, to our knowledge, has never been used in any study on sport fan aggression. To date, the most common research strategies used to explain the causes of football fan aggression take the form of either an epidemiological approach or a survey approach (for a review on sport riots see [4]).

The procedure of the epidemiological approach is to group many violent sport incidents together and then relate its occurrence to external factors, which were going on around the same time of the violent incidents. This approach has signaled some important factors that may be related to football violence. For example, it has been found that the occurrence of weekly hooliganism increased when these incidents were preceded by media coverage on hooliganism, when unemployment was on the rise, and when there was more aggressive play on the pitch (four Dutch premier league football seasons, *n* = 196) [5]. In line with this latter result, a study among 297 Israeli football teams revealed that higher levels of player violence were related to higher levels of spectator violence [6]. On the other hand, police suppression did not seem to affect hooliganism in the Dutch premier league [5]. However, some policing activity may reduce violence. For example, in Sweden, the absence of specialized police units that monitor hooligan groups lead to a threefold increase in violent incidents [7]. Apart from these factors it also appears that frustration among supporters is associated with violence. For example, the number of times supporters throw objects onto the field during matches of the highest Swedish football league was associated with a drop in the team’s position in the ranking [8]. They showed that, on average, a one position drop was related to an increase of as much as 6% of throwing behavior, which is a large effect considering that positions tend to change frequently in a football season. Interestingly, absolute ranking position did not influence throwing behavior. Furthermore, it seems that violence is not only committed in or around the stadium but also at home. When extending our scope to American football, it appears that home related violence by men against their wives and girlfriends increased by as much as 10% when their team unexpectedly experienced a big loss (family violence data 12 year period of 750 city and county police agencies) [9]. Similarly, in Scottish football, domestic violence increased by 36% in the local area when the two big rival teams played (Celtic vs. Rangers), although an increase in domestic violence due to an unexpected loss was limited to only few matches [10]. Finally, English police reports revealed that when the English national football team lost a match during a FIFA world cup this was accompanied by a 38% increase in domestic violence [11].

The survey approach has yielded a different perspective on football related violence, since this approach can explain violence better at the individual level. This research has mainly focused on the identity of hooligans and sees this identity as the main factor explaining the occurrence of violence. For example, a qualitative analysis of semi-structured interviews in the field (*n* > 400) has described that central to a hooligans’ identity are (i) excitement and pleasurable emotional arousal, (ii) hard masculinity, (iii) territorial identifications, (iv) individual and collective management of reputation, (v) a sense of solidarity and belonging, (vi) and representations of sovereignty and autonomy [12]. However, it may be that these previous findings are specific for hooligans, as other researchers have found varying relationships between identity and violence in non-hooligan samples. For example, identification with the role of sport fan was unrelated to trait aggression in a sample of psychology students (*n* = 70) [13]. However, a study that administrated questionnaires in a bar of football fans (*n* = 109) found that social identity (i.e., identification with their own team) was the best predictor of self-reported physical aggression and superior to loss of private and public self-awareness [14]. Finally, in a sample of Dutch fans and nonfans, it appeared that Dutch football fans (*n* = 60) scored higher on psychopathy than Dutch nonfans (*n* = 43), but were not different on an assault scale [15].

We seek to complement the epidemiological and survey approach to football fan aggression with a controlled laboratory study, which should be especially helpful in identifying the causes of football fan aggression. Surprisingly, to our knowledge, there are no studies to date that have investigated football fan aggression in a controlled laboratory setting. Perhaps this is because a laboratory setting can be viewed as a poor artificial replication of the dynamic nature of a riot and that this research design does not permit to investigate organized supporter violence independent of a match [16]. Although this critique is valid, a standardized experimental protocol has benefits that the epidemiological and survey approach do not have and can thus complement the other approaches. For example, in a controlled laboratory study, it is possible to isolate key variables and avoid biases inherent to self-reports that are common in surveys.

Therefore, we designed a study in which football fans could physically aggress against a fan from a rival team according to the hot sauce paradigm [17], an ecologically valid and ethical lab procedure to measure real overt aggression [18]. Participants first saw a match summary in which their favorite team lost against their most important rival. Next, they were given the opportunity to administer hot sauce to a sample that a football supporter from their most important rival had to consume. From an intervention perspective, media exposure is perhaps one of the most promising factors to be addressed, since it has been shown that previous media coverage on football violence may increase upcoming violent incidents [5]. A relatively easy intervention would be to show videos of fans expressing their respect for their rival before a match. Especially after a lost match, fan aggression and anger may be reduced, since more of the fans’ social status may be left intact. To investigate this, before watching a video of a match, participants saw a video in which fans of their rival team commented in a neutral, negative, or positive manner on their favorite team. We hypothesized that positive comments would reduce aggression and that negative comments would increase aggression, compared to the neutral comments.

Furthermore, we advanced a model of predictors for fan aggression, which allowed us to examine whether factors identified by the epidemiological approach (personal characteristics such as age and fandom) were related to the expression of aggression as well as mood changes and match appraisal. Finally, we examined whether participants’ levels of testosterone and cortisol affected aggression since it has been shown that fans watching a football match have higher testosterone and cortisol levels compared to a control day (*n* = 50) [19]. A tentative hypothesis could be that these hormonal changes are accompanied by higher levels of aggression. Moreover, it is possible that differences in basal testosterone and basal cortisol might be associated with aggression. In particular, there is some evidence revealing that basal testosterone is related to observed and self-reported aggression, although effect sizes are small [20]. Furthermore, lower levels of basal cortisol in boys have been related to various forms of proactive aggression [21–23]. Therefore, in an exploratory vein, we examined whether basal testosterone and basal cortisol might be associated with greater levels of aggression, thereby examining the hormonal basis of aggression in the context of football fan aggression.

## Methods and Materials

### Participants

A total of 74 male football fans completed the study (18–35 years, *M* = 20.84, *SD* = 3.17). Only self-proclaimed fans from the local football team (AFC AJAX) were included in this study since the videos participants saw were tailored to AFC AJAX fans. The sample consisted of 67 students and 7 non-students. On average, participants were fan of their team for 14.03 years (*SD* = 5.53), 19% of participants belonged to a supporter club (*n* = 14), participants saw at least one match of their team on television per month (*M* = 3.66, *SD* = 1.17), 23% of participants had a season pass to visit their team when they played (*n* = 17), 15% of participants indicated to be part of the “hard core” fans (*n* = 11), and 14% of participants went to one or more away matches of their team per month (*n* = 10). Participants were recruited by distributing flyers and approaching people at the VU University in Amsterdam and nearby football clubs. Volunteers were excluded from participation if they studied psychology due to their possible knowledge and experience of studies in our lab, and were excluded if they had psychological or physical medical problems that could influence their hormonal or psychological response to the manipulation (e.g., depression or use of corticosteroids, [24]). Eight participants failed to complete the study: seven participants accidently administrated the hot sauce onto their clothes due to a malfunctioning syringe and for one participant the software crashed. Participants were informed that they should have eaten two hours before the start of the experiment and that on the day of the experiment they should not consume alcohol. They were also informed that up to two hours before the study they should not take food or drinks containing caffeine (e.g., coffee, chocolate). On the day before the experiment participants were informed to go about their normal habits, and they were instructed not to go out until late, to not drink a lot of alcohol, to not engage in any extreme physical activity, and to get a normal night’s rest. Participants received €10 for their participation. This study was approved by the Ethics Committee of the Faculty of Psychology and Pedagogy of the VU University Amsterdam (Vaste Commissie Wetenschap en Ethiek van de Faculteit der Psychologie en Pedagogiek: VCWE; reference: E1306LM).

### Procedure

**Fig 1:**
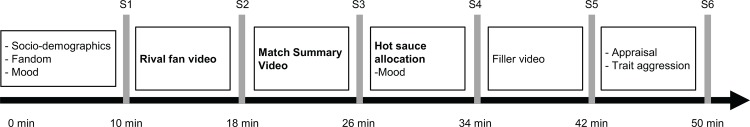
Timeline of the experiment. S = saliva sample.

The experiment was conducted in the laboratory of psychology at the VU University Amsterdam (the Netherlands). See Fig. 1 for the timeline of the experiment. Upon arrival, participants were informed about the general outline of the study and signed an informed consent form. Participants were escorted to their cubicle where they completed the study. Instructions were given to participants through the online survey program Qualtrics. Next, participants filled in the questionnaires on socio-demographics, level of fandom, and their current mood. Then Qualtrics assigned participants randomly (with even distribution to condition) to one of the conditions, ensuring that the experimenter was blind to the condition to which the participant was assigned. The participants were equally assigned to conditions: 26 fans participated in the positive rival fan video condition, 24 fans participated in the negative rival fan video condition, and 24 fans participated in the neutral rival fan video condition. Subsequently, the participants saw one of the rival fan videos (see section Videos). After this video the participants saw the match summary video in which their team lost against their biggest rival. Next, participants completed the hot sauce paradigm and filled in a questionnaire on their mood and a questionnaire assessing match appraisal. Participants then saw a filler video, and afterwards they filled in the questionnaires on trait aggression. Finally, participants were paid and debriefed.

### Videos

A total of 3 videos were shown to participants:

- **Rival fan video**. Duration 3:50 min. This video consisted of three actors impersonating football fans of their biggest rival football club (Feyenoord) who provided in interview sessions either positive, negative, or neutral comments about AFC Ajax fans. Examples of positive comments were: “Ajax has a great youth training program” and “I respect Ajax”. Examples of negative comments were: "None of the Ajax fans are real football supporters, they're all fake " and “They think they are so great but achieve nothing”. Examples of neutral comments were: “The nice thing about football is that you can watch the match with friends and beer” and “Football is fun, because there's strategy involved.”
- **Match summary video**. Duration 5:36 min [25]. Match summary between Feyenoord and AFC Ajax on January 29th, 2012 (a year before the experiment) in Feyenoord’s home-stadium ‘De Kuip’. The result of this match was a 4–2 loss for AFC Ajax. This particular match was chosen because Feyenoord fans appear cheering extensively, which makes the atmosphere reflected in the video especially negative for AFC Ajax fans. 74.30% of participants had watched the match live and 63.50% of participants indicated that they remembered the outcome of the match.
- **Filler video**. Duration 8:08 min [26]. This filler video was a neutral/positive documentary on the merger of two Dutch amateur football clubs.

### Hot sauce allocation

To measure the participants’ physical aggression they completed the hot sauce allocation task [17] just after having watched the match video in which their favorite team lost. Participants had to put hot sauce (Tabasco Original Red Sauce) into a cup using a prepared syringe filled with the sauce. They were told that the amount of hot sauce they put into the cup would later be put into a dish and administered to a fan of the opposing team to which they just lost (Feyenoord fan). In reality, nobody consumed the hot sauce which was explained in the debriefing. The maximum amount of hot sauce they could administer was on average 11.81 g (*SD* = 0.68; there were small differences in how much hot sauce each syringe contained). To ensure that participants were aware of the spiciness of the hot sauce, they were instructed to taste a sample before administering it.

### Questionnaires

#### Fandom

To measure the level of participants’ fandom of AFC AJAX they answered several questions regarding their activities related to football. Participants were grouped into two groups based on their answers: committed fans (*n* = 40) and non-committed fans (*n* = 34). Participants included in the committed fan group complied with one of the following criteria: (i) visit one or more away matches per month, (ii) have a season pass, (iii) be a member of a fan club, (iv) form part of the hard core fans, (v) watch all their team’s matches on television.

#### Mood

To measure positive and negative affect participants filled in the PANAS scale [27] before the rival fan video and after the match video. This questionnaire consists of 10 negative adjectives (e.g., upset; before video: α = 0.80; after video: α = 0.86) and 10 positve adjectives (e.g., active; before video: α = 0.84; after video: α = 0.85). They had to indicate for each adjective the extent to which it described their feelings at this moment; we used a scale ranging from 1 “absolutely not” to 5 “very strongly”. Together with the PANAS adjectives, participants also filled in six adjectives measuring state anger [28]. Item examples are: irritated, hostile, and angry (before video: α = 0.91; after video: α = 0.89). The item ‘agitated’ was not correctly translated and therefore removed from the scale.

#### Appraisal

To measure how participants perceived the match they answered five questions on a five point Likert scale ranging from 1 (not at all) to 5 (a lot). They rated the degree to which they felt the match was stressful and frustrating and they indicated how much they thought their own team had difficulty with the match, how well they played, and how much they struggled with the match. Participants were also asked to rate the extent to which they thought eight different factors contributed to the outcome of the match. They indicated their answer on a five point Likert scale ranging again from 1 (not at all) to 5 (a lot). The factors were the following: (i) referee, (ii) performance players opposing team, (iii) performance players own team, (iv) their own level of fandom, (v) chance, (vi) fan behavior opposing team in stadium, (vii) state of the field, (viii) atmosphere in the stadium. There were no differences in how participants scored the appraisal items according to the rival fan video condition they were in (*F*_26,120_ = 0.746, *p* = 0.805, η_p_^2^ = 0.139).

#### Trait Aggression

To assess participants’ trait aggression they filled in the 12 item short form of the Buss-Perry Aggression Questionnaire [29]. This scale consisted of four factors with three items measuring each factor: (i) physical aggression (e.g., “given enough provocation, I may hit another person”; α = 0.70), (ii) verbal aggression (e.g., “I can’t help getting into arguments when people disagree with me”; α = 0.67), (iii) anger (e.g., “I have trouble controlling my temper”; α = 0.78), and hostility (e.g., “other people always seem to get the breaks”; α = 0.79). There were no differences in how participants scored these subscales according to the rival fan video condition they were in (*F*_8,138_ = 0.695, *p* = 0.695, η_p_^2^ = 0.039).

### Hormonal measurements

During the study participants provided six times 2 ml of saliva by passively drooling into a small tube for the measurement of their cortisol and testosterone levels. The first saliva sample was taken before the rival fan video (+10 min), the second sample before the match video (+18min), the third sample before the hot sauce administration (+26 min), the fourth sample before the filler video (+34 min), the fifth sample before filling in the final questionnaires (+42 min) and the sixth sample before debriefing (+50 min). The samples were frozen at −20°C and were sent frozen to the laboratory of Biological Psychology at the Dresden University of Technology. To determine testosterone the laboratory in Dresden used an expanded range salivary testosterone enzyme-immunoassay kit (cat. nu 1–2402) from Salimetrics (Suffolk, UK) and to determine cortisol they used a luminescence immunoassay kit (RE62011) from IBL International (Hamburg, Germany). The intra and inter assay coefficients are below 10% and 12% for testosterone and below 6% and 8% for cortisol. Hormonal values were log transformed because they did not follow a normal distribution. Since time intervals between samples were all 8 minutes we calculated the total hormonal level change by subtracting the mean of post samples 2–6 from the baseline sample. One participant did not supply enough saliva to assay hormonal levels. Hormonal values that differed more than three standard deviations from the mean were not removed from the analyses and their removal did not change the statistical conclusions of the analyses.

### Statistical analyses

For all the analyses concerning the hot sauce allocation we used the percentage of total hot sauce administered, and not the total amount of hot sauce administered in grams, because there were small differences in how much hot sauce each syringe contained when prepared for the participant (*M* weight syringe = 20.39 g, *SD* = 0.74). To investigate the effect of watching the match summary on mood and hormonal levels we performed repeated measures ANOVAs. As repeated measures we included Moment as within subject variables (pre vs. post-match mood scores or the six hormonal samples) and included Condition (pos., neg., neut. rival fan video) as a between subject variable. Post hoc *t*-tests (paired or independent) were performed if there was a significant interaction between Moment and Condition. The Greenhouse-Geisser correction was applied if the assumption of sphericity was violated.

To investigate what predictors were related to the amount of hot sauce administered we performed Pearson correlations for each individual predictor separately (for the predictor fandom we performed an independent *t*-test). For all correlations concerning hormones we performed partial correlations, controlling for starting time of the experiment, to control for variation in circadian rhythm (start time: *M* = 14:03, *SD* = 01:40, Min = 10:34, Max = 17:02). Because there is not much existing research that could inform us about the predictors of football fan aggression, we considered the analysis novel and largely exploratory. Therefore, we decided not to include multiple predictors in the same analyses for the following reasons: (i) to avoid problems of multicollinearity, (ii) it is theoretically unclear which predictors have to be added in the same regression analysis (i.e., to control for which factors), and (iii) by providing the raw data future research is stimulated to disentangle which unique factors contribute to fan aggression in football. Furthermore, we did not correct for multiple comparisons as this procedure reduces power which may lead to the rejection of important true findings [30,31] and may increase publication bias [32]. To illustrate this, if the Bonferroni correction for multiple comparisons was applied to the analyses in this article, only *p*-values ≤ 0.002 (or correlations ≥ 0.36) would be statistically significant (*n* = 74, 26 comparisons). Thus, none of the reported correlations in this article would be statistically significant. Instead of correcting for multiple comparisons, we reported standardized effect sizes to assess behavioral importance [32]. In the supporting information, we have included a complete correlation matrix of all the predictors (S1 Table), a table with the main outcome variables according to rival fan feedback (S2 Table), and the full data matrix (S1 Data Matrix). For the statistical analysis SPSS 22.0 was used and *p* values ≤ 0.05 (two tailed) were considered statistically significant.

## Results

### Rival fan video

#### Hot sauce administration

The rival fan videos did not affect the amount of hot sauce administered (*F*_2,71_ = 0.892, *p* = 0.414, η_p_^2^ = 0.025). On average, participants administered 53.06% (SD = 38.60) of the hot sauce contained in the syringe (*M* = 6.23 g, *SD* = 4.59) and 33.80% of participants (*n* = 25) administered all of the hot sauce.

#### Mood

Negative and positive mood were not affected by the rival fan video they watched (respectively: *F*_2,71_ = 0.919, *p* = 0.404, η_p_^2^ = 0.025; *F*_2,71_ = 0.104, *p* = 0.902, η_p_^2^ = 0.003). However, irrespective of the rival fan video, negative mood did increase after having watched the match (*F*_1,71_ = 22.10, *p* < 0.001, η_p_^2^ = 0.237). The average individual increase in negative mood was 32.75% (*SD* = 58.84). Positive mood did not change after having watched the match (*F*_1,71_ = 1.270, *p* = 0.264, η_p_^2^ = 0.018).

State anger was affected by the rival fan video they watched (*F*_2,71_ = 3.133, *p* = 0.050, η_p_^2^ = 0.081). As expected, in all conditions, state anger increased after the match (positive: *t*_25_ = −3.310, *p* = 0.003, *dz* = 0.649; negative: *t*_23_ = −4.839, *p* < 0.001, *dz* = 0.988; neutral: *t*_23_ = −5.124, *p* < 0.001, *dz* = 1.046), and there were no baseline differences in state anger between conditions (all *p* ≤ 0.615). However, state anger increased differently between conditions, since state anger was lower in the positive rival fan video condition after the match than in the negative and neutral rival fan video conditions (respectively: *t*_48_ = −2.160, *p* = 0.036, *d* = 0.609; *t*_41.63_ = −2.283, *p* = 0.028, *d* = 0.650). State anger after the match was not different between the negative and neutral rival fan conditions (*t*_46_ = 0.259, *p* = 0.797, *d* = 0.075). The average individual increase in state anger in the positive rival fan video condition was 40.98% (*SD* = 57.33), in the neutral rival fan video condition it was 93.24% (*SD* = 89.47), and in the negative rival fan video condition it was 81.25% (*SD* = 82.83).

#### Hormones

Testosterone and cortisol levels did not change differently across rival fan video conditions (respectively: *F*_7.43,256.28_ = 0.723, *p* = 0.661, η_p_^2^ = 0.021; *F*_6.15,212.05_ = 0.925, *p* = 0.480, η_p_^2^ = 0.026). Irrespective of the rival fan video condition, testosterone and cortisol levels did not change after having viewed the match (respectively: *F*_3.71,256.28_ = 0.409, *p* = 0.788, η_p_^2^ = 0.06; *F*_3.07,212.05_ = 0.885, *p* = 0.452, η_p_^2^ = 0.13).

### Predictors for football fan aggression

**Fig 2:**
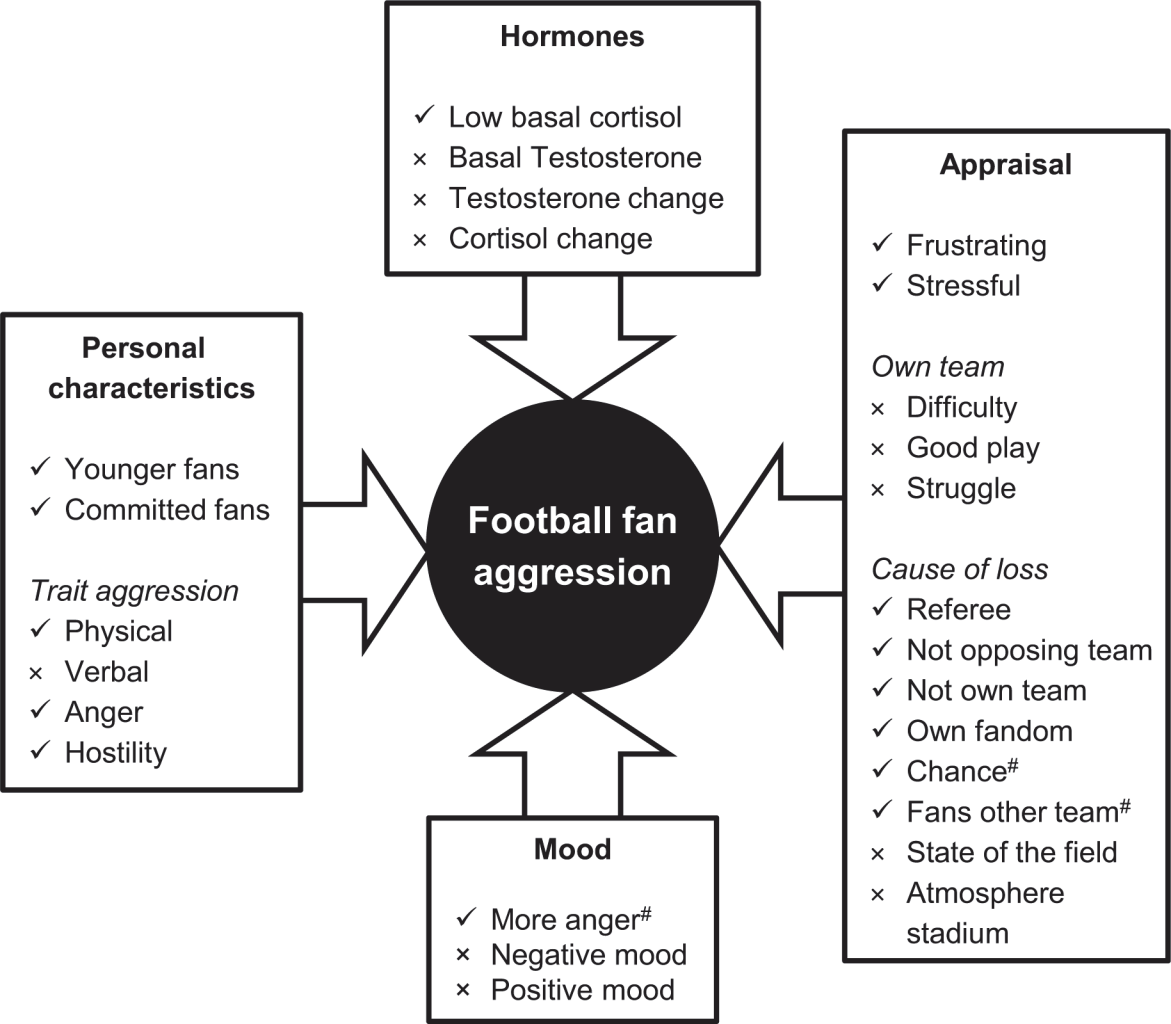
Predictors for football fan aggression. The relationship between participants’ personal characteristics and situational factors with physical aggression (amount of hot sauce). ✓ = significant predictor (*p* < 0.05), × = no significant predictor (*p* > 0.10), ^#^ = marginally significant predictor (*p* < 0.10).

See Fig. 2 for a result summary of the predictors for football fan aggression.

#### Personal characteristics

The younger the participants were the more hot sauce they administered (*r*_74_ = −0.238, *p* = 0.041). The amount of hot sauce administered differed between committed and non-committed fans (*t*_72_ = 2.350, *p* = 0.021, Cohen’s *d* = 0.55). Committed fans administered on average 62.50% of the total amount of hot sauce (*SD* = 39.74) and non-committed fans administered 41.96% of the total amount of hot sauce (*SD* = 34.57). The more hot sauce participants administered the higher they scored on physical aggression (*r*_74_ = 0.345, *p* = 0.003), anger (*r*_74_ = 0.342, *p* = 0.003), and hostility (*r*_74_ = 0.268, *p* = 0.021). However, the amount of hot sauce administered was not related to verbal aggression (*r*_74_ = 0.064, *p* = 0.591).

#### Mood

Marginal evidence revealed that participants administered more hot sauce to the extent that they had experienced an increase in anger (*r*_74_ = 0.224, *p* = 0.055). However, the amount of hot sauce administered was not related to changes in positive mood (*r*_74_ = 0.150, *p* = 0.202) and negative mood (*r*_74_ = 0.131, *p* = 0.265).

#### Match appraisal

The more hot sauce participants administered the more they perceived the match as frustrating (*r*_74_ = 0.264, *p* = 0.023) and stressful (*r*_74_ = 0.248, *p* = 0.033). However, the amount of hot sauce administered was not related to how much participants perceived that their own team had difficulty with the match (*r*_74_ = 0.034, *p* = 0.772), how well they played (*r*_74_ = −0.005, *p* = 0.968), or how much they struggled with the match (*r*_74_ = −0.081, *p* = 0.494). The perceived cause of the match result can be an important motivator of aggression. Results revealed that greater levels of hot sauce were administered to the degree that the referee and their own fandom were blamed for the match result (respectively: *r*_74_ = 0.241, *p* = 0.039; *r*_74_ = 0.242, *p* = 0.038). Less hot sauce was administered to the degree that the players’ performance of the opposing team and the players’ performance of own team were blamed for the match result (respectively: *r*_74_ = −0.267, *p* = 0.022; *r*_74_ = −0.295, *p* = 0.011). Also, there were marginal associations between the amount of hot sauce and “chance” and fan behavior of the opposing team as causes for the match result (respectively: *r*_74_ = 0.217, *p* = 0.063; *r*_74_ = 0.203, *p* = 0.083). Finally, the amount of hot sauce administered was neither related to perceiving the state of the field nor the atmosphere in the stadium as causes for the match result (respectively: *r*_74_ = 0.058, *p* = 0.625; *r*_74_ = 0.101, *p* = 0.393).

#### Hormones

The amount of hot sauce administered was not related to the total testosterone change (*pr*_70_ = −0.146, *p* = 0.222) nor to the total cortisol change (*pr*_70_ = 0.083, *p* = 0.490). The lower the participants’ cortisol level at baseline the more hot sauce they administered (*pr*_70_ = −0.281, *p* = 0.017). However, baseline testosterone was not related to the amount of hot sauce administered (*pr*_70_ = −0.027, *p* = 0.824).

## Discussion

The results from this study do not support the idea that football fan aggression will be reduced by showing fans videos of supporters from the opposing team expressing respect for their supporter group. An explanation for this null finding is that watching your own team lose is a too powerful situational influence that is not easily mitigated by information from external sources. Another explanation could be that the fans had trouble believing the videos. However, this explanation is not in line with the mood changes we observed, as there was a tendency for participants to feel less angry when having watched the videos in which supporters of the opposing team commented positively on their supporter group than when they saw videos in which these supporters commented negatively or neutrally on their supporter group. It could thus be that although the videos did change to some extent how they felt, it did not reduce any real aggression. Indeed, to reduce physical aggression may be complex as it is the result of a complex psychological process that, apart from mood, also depends on the appraisal of the situation and individual differences in personality [33].

A second goal of the study was to examine a model that explores the major predictors of fan aggression. At the outset, we should acknowledge that these analyses are exploratory, and that caution has to be taken in interpreting this model as causality could not be established in this study, nor could this study clearly assess the relative importance of the factors explaining football fan aggression. Indeed, the model was informed by past epidemiological and survey research, and now tested in a controlled experimental setting, which can be seen as both a strength and a weakness; key variables may be better isolated, but it is very difficult to replicate the social aspects of football fan aggression. While exploratory, the findings uncovered several important predictors of football fan aggression and patterns that were generally quite consistent with past survey research in particular. Regarding personal characteristics, previous research has found that young committed fans report to commit more violence around football matches [2,3]. In line with this, this study found that especially young, committed fans were more aggressive. A possible explanation for this finding is that men in their early twenties are more aggressive than men in their later twenties because they generally exhibit greater tendencies to impulsivity [34], and more impulsivity is related to more physical aggression when provoked [35]. Furthermore, for committed fans more of their social status was at stake and thus when their team lost they may have been more frustrated, and according to the frustration-aggression hypothesis, this may have fueled more aggression [36]. Also, trait aggression was related to more aggression which adds further credence to the idea that the administration of the hot sauce paradigm provides a specific yet valid measure of aggression. It also shows that the paradigm was correctly implemented, since previous research has shown that higher scores on trait aggression resulted in more hot sauce administration [17].

Our findings on the relationship between appraisal and aggression are perhaps most interesting from an intervention perspective. Results showed that it does matter how fans appraised the outcome of the match. One of the most important contextual factors appeared to be in how much fans blamed the result to external factors such as the referee and to internal factors such as performance of their own team. Aggression was higher when the referee was blamed and aggression was lower when the performance of the own team was blamed for the match result. These results thus reflect that when the match result was seen as unfair, i.e., caused by the referee and not caused by own team performance, aggression increased. This conclusion is supported by a wealth of evidence showing that aggression increases when justice is perceived to be low. For example, indirect aggression (e g., obscene gestures, silent treatment) by students towards their teachers increases when perceived procedural justice decreases [37,38]. Also, in the workplace, more indirect aggression (e.g., working slower or stealing supplies) has been related to lower views of perceived justice [39].

The evidence described above suggests the importance of the question: How can we increase the perceived fairness of a match? Clearly, in various discussions outside of science, this is a classic and ongoing issue. What might be the ways in which referee decisions become more free from error and bias, or perceived error and bias? Perceptions of justice and fairness are in the eyes of the spectator, and there is little doubt that many of the perceptions might be colored by a strong desire for their team to win, or at least not to lose. One important source for potential improvement would be to use technical innovations by which decisions can be more factual. For example, the use of hawk eye technology by which it can be accurately assessed whether the ball passed a line or not would be helpful—not only because it is accurate, but because it is likely to be perceived as free from error *and* bias. Alternatively, for some other critical decisions, for example, those requiring human judgment (such as judgments of fouls or offside) it is perhaps possible for important matches to have small committees (consisting of three or five members) to make a decision on the basis of majority vote. It seems important to evaluate such solutions not only in terms of increasing the accuracy of referee decisions but also in terms of promoting fairness of the procedure by which a decision is made. As alluded to earlier, in social settings (such as schools or organizations), there is considerable evidence showing that so-called procedural justice—the fairness of the procedure by which decisions are made—is key to understanding people’s tendencies to accept and support decisions, rather than to protest or engage in more destructive forms of behavior [40,41].

This study also assessed whether the hormones cortisol and testosterone were related to the expression of aggression. Although previous studies have found that cortisol and testosterone levels were higher when fans were watching a live match with fellow supporters [19,42], we did not find evidence for any change in these hormones over time. This suggests that fellow supporters, a live match, and a non-laboratory setting are needed to increase arousal sufficiently to produce cortisol or testosterone changes. Interestingly, we did find that fans with lower baseline cortisol levels administered more hot sauce to the rival football supporter. To our knowledge, the negative relationship between basal cortisol and actual aggressive behavior has not been documented in the domain of football, or more generally, rival intergroup relations. At the same time, similar patterns have been observed in other domains, where aggressiveness is often measured by ratings by others, and sometimes self-ratings. For example, research has shown that the more teachers report aggressive symptoms in their students with conduct disorder, the lower basal cortisol levels these boys (6–12 years) have [21]. Also, in a sample of clinically-referred boys (7–12 years), lower cortisol levels were related to more aggression as rated by their peers [22]. Furthermore, low cortisol levels during preadolescence (10–12 years) were related to more aggressiveness at middle adolescence (15–17 years), which was mediated by low self-control [23]. One might thus speculate that low cortisol levels seem related to proactive aggression, which is controlled by and related to frequent anti-social behavior, and less strongly related—or not related—to reactive aggression, as this form of aggression is impulsive with high levels of arousal and is related to high levels of cortisol [43]. At the same time, some have argued the reverse. For example, in a student sample, low basal cortisol levels predicted more costly punishment in the public goods game, and this costly punishment can be interpreted as a form of reactive aggression accompanied by impulsivity under low arousal conditions [44]. The present findings may further fuel this debate, as participants’ arousal was probably relatively low, since there was no detectable hormonal change, and the allocation of hot sauce can be viewed as either proactive or reactive aggression. It may be proactive—simply because the other is a supporter of a rival team—or it may be reactive, if it is triggered to some degree by referee decisions. Thus, while the present research findings add to the robustness of the link between low basal cortisol and aggression, future research is needed to uncover the precise meaning of that connection.

We hope that the results from this study will signal to future research the most promising causes of sport aggression when studying it in controlled laboratory conditions. Of special interest is the finding showing that aggression increased when the cause for the lost match was blamed on the referee. To consolidate this finding, future research could experimentally manipulate the possibility to attribute the match result to the referee. Also of special interest is the finding that fans with low basal cortisol levels displayed more aggression, as this suggests that at least part of the aggression was proactive and related to anti-social behavior. A recommendation for future research would be to extend the methodology to a wider array of football matches since epidemiological studies have shown that aggression may be amplified when matches result in a drop in ranking and when suffering a large defeat to a weaker team.

## Supporting Information

S1 Table Correlation matrix of all the variables predicting hot sauce administration. Correlations are Pearson’s *r*. * = *p* ≤.05, ** = *p* ≤.01.
(DOCX)

S2 Table Means and standard deviations of the outcome variables according to rival fan video condition. (DOCX)

S1 Data Matrix Data matrix in SPSS format (.sav) used for the analyses in this study. (SAV)
