# Supplementary material for: Football Fan Aggression: The Importance of Low Basal Cortisol and a Fair Referee
Source: PLoS One. 2015 Apr 6;10(4):e0120103. doi: 10.1371/journal.pone.0120103 (PMC4386810; doi:10.1371/journal.pone.0120103)
Supplement: S1 Table — Correlations are Pearson’s r. * = p ≤.05, ** = p ≤.01. (DOCX) [file pone.0120103.s001.docx]

**Table S1. Correlation matrix of all the variables predicting hot sauce administration.** Correlations are Pearson’s *r*. * = *p* ≤.05, ** = *p* ≤.01.

|  |  | 1 | 2 | 3 | 4 | 5 | 6 | 7 | 8 | 9 | 10 | 11 | 12 | 13 | 14 | 15 | 16 | 17 | 18 | 19 | 20 | 21 | 22 | 23 | 24 | 25 | 26 |
| --- | --- | --- | --- | --- | --- | --- | --- | --- | --- | --- | --- | --- | --- | --- | --- | --- | --- | --- | --- | --- | --- | --- | --- | --- | --- | --- | --- |
| 1 | Hot sauce administered (%) |  |  |  |  |  |  |  |  |  |  |  |  |  |  |  |  |  |  |  |  |  |  |  |  |  |  |
| 2 | Age (yrs.) | -.238^*^ |  |  |  |  |  |  |  |  |  |  |  |  |  |  |  |  |  |  |  |  |  |  |  |  |  |
| 3 | Fandom (committed or not) | -.278^*^ | -.056 |  |  |  |  |  |  |  |  |  |  |  |  |  |  |  |  |  |  |  |  |  |  |  |  |
|  | **Mood** |  |  |  |  |  |  |  |  |  |  |  |  |  |  |  |  |  |  |  |  |  |  |  |  |  |  |
| 4 | Anger Change (post-pre) | .224 | -.047 | -.132 |  |  |  |  |  |  |  |  |  |  |  |  |  |  |  |  |  |  |  |  |  |  |  |
| 5 | Positive Mood Change (post-pre) | .150 | .007 | -.057 | -.213 |  |  |  |  |  |  |  |  |  |  |  |  |  |  |  |  |  |  |  |  |  |  |
| 6 | Negative Mood Change (post-pre) | .131 | .091 | -.076 | .678^**^ | -.343^**^ |  |  |  |  |  |  |  |  |  |  |  |  |  |  |  |  |  |  |  |  |  |
|  | **Trait Aggression** |  |  |  |  |  |  |  |  |  |  |  |  |  |  |  |  |  |  |  |  |  |  |  |  |  |  |
| 7 | Physical Aggression | .345^**^ | -.058 | -.258^*^ | .081 | -.044 | .098 |  |  |  |  |  |  |  |  |  |  |  |  |  |  |  |  |  |  |  |  |
| 8 | Verbal Aggression | .064 | .011 | .035 | .107 | .087 | -.020 | -.028 |  |  |  |  |  |  |  |  |  |  |  |  |  |  |  |  |  |  |  |
| 9 | Anger | .342^**^ | -.038 | -.289^*^ | .316^**^ | -.068 | .088 | .449^**^ | .469^**^ |  |  |  |  |  |  |  |  |  |  |  |  |  |  |  |  |  |  |
| 10 | Hostility | .268^*^ | .012 | -.185 | .256^*^ | -.207 | .179 | .570^**^ | .135 | .545^**^ |  |  |  |  |  |  |  |  |  |  |  |  |  |  |  |  |  |
|  | **Appraisal** |  |  |  |  |  |  |  |  |  |  |  |  |  |  |  |  |  |  |  |  |  |  |  |  |  |  |
| 11 | Own team struggled with match | -.081 | .057 | .174 | .041 | -.058 | .220 | -.027 | .030 | -.094 | -.204 |  |  |  |  |  |  |  |  |  |  |  |  |  |  |  |  |
| 12 | Frustrating to watch | .264^*^ | .001 | -.155 | .321^**^ | -.006 | .126 | -.015 | .050 | .098 | -.014 | .137 |  |  |  |  |  |  |  |  |  |  |  |  |  |  |  |
| 13 | How well they played | -.005 | .011 | -.083 | .029 | -.019 | .078 | .118 | .016 | .098 | .120 | -.337^**^ | -.019 |  |  |  |  |  |  |  |  |  |  |  |  |  |  |
| 14 | Stressful watching | .248^*^ | .127 | -.162 | .344^**^ | .183 | .136 | .111 | .040 | .277^*^ | .067 | .191 | .562^**^ | -.148 |  |  |  |  |  |  |  |  |  |  |  |  |  |
| 15 | Difficulty with match | .034 | -.052 | -.028 | -.255^*^ | .059 | -.083 | .186 | -.164 | -.213 | -.038 | .116 | -.041 | .002 | -.059 |  |  |  |  |  |  |  |  |  |  |  |  |
|  | **Cause of loss** |  |  |  |  |  |  |  |  |  |  |  |  |  |  |  |  |  |  |  |  |  |  |  |  |  |  |
| 16 | Chance | .217 | .071 | -.145 | .018 | .163 | -.159 | .012 | .037 | .116 | -.055 | -.043 | .177 | .062 | .229^*^ | .067 |  |  |  |  |  |  |  |  |  |  |  |
| 17 | State of the field | .058 | -.026 | .119 | .269^*^ | -.122 | .099 | .027 | .141 | .241^*^ | .173 | -.041 | -.038 | .245^*^ | .136 | -.184 | -.003 |  |  |  |  |  |  |  |  |  |  |
| 18 | Atmosphere in the stadium | .101 | .043 | .025 | -.065 | .280^*^ | -.179 | -.088 | .022 | .013 | .021 | -.178 | .221 | .103 | .227 | .046 | .237^*^ | .033 |  |  |  |  |  |  |  |  |  |
| 19 | Fan behavior opposing team | .203 | -.052 | -.060 | .044 | .076 | -.021 | -.018 | .038 | .146 | .159 | -.131 | .256^*^ | -.056 | .198 | .113 | .003 | .068 | .726^**^ |  |  |  |  |  |  |  |  |
| 20 | Referee | .241^*^ | .175 | -.235^*^ | .062 | .152 | -.044 | -.035 | .168 | .161 | -.088 | .076 | .222 | -.038 | .232^*^ | -.115 | .440^**^ | .177 | .110 | .105 |  |  |  |  |  |  |  |
| 21 | Players’ performance opposing team | -.267^*^ | .057 | .304^**^ | -.323^**^ | .104 | -.191 | .068 | -.033 | -.261^*^ | -.168 | .205 | -.175 | .103 | -.171 | .029 | .017 | -.117 | -.052 | -.290^*^ | -.118 |  |  |  |  |  |  |
| 22 | Players’ performance own team | -.295^*^ | .171 | .277^*^ | -.249^*^ | .134 | -.268^*^ | .064 | -.015 | -.234^*^ | -.145 | -.015 | -.045 | -.019 | -.034 | .118 | -.022 | -.148 | -.015 | -.265^*^ | -.076 | .741^**^ |  |  |  |  |  |
| 23 | Own fandom | .242^*^ | -.017 | -.133 | .073 | -.085 | .003 | .130 | -.138 | .234^*^ | .298^**^ | -.099 | .110 | .080 | .153 | -.070 | -.127 | .069 | .088 | .334^**^ | -.060 | -.305^**^ | -.347^**^ |  |  |  |  |
|  | **Hormones** |  |  |  |  |  |  |  |  |  |  |  |  |  |  |  |  |  |  |  |  |  |  |  |  |  |  |
| 24 | Testosterone baseline (log) | -.029 | -.045 | .133 | .044 | .045 | -.105 | -.136 | .035 | -.044 | -.140 | .050 | .171 | -.108 | .069 | .055 | -.007 | .032 | .145 | .060 | .188 | -.153 | -.013 | -.078 |  |  |  |
| 25 | Cortisol baseline (log) | -.283^*^ | .241^*^ | .218 | -.220 | -.115 | -.086 | -.161 | -.064 | -.209 | -.300^**^ | .074 | .042 | -.017 | -.025 | -.021 | -.213 | -.134 | -.348^**^ | -.323^**^ | .016 | .200 | .184 | .033 | .157 |  |  |
| 26 | Testosterone change (log) | -.149 | .121 | -.031 | -.123 | .044 | -.176 | -.001 | .096 | -.022 | -.056 | -.054 | -.062 | .149 | -.085 | -.243^*^ | .082 | .034 | -.105 | -.242^*^ | .058 | .244^*^ | .221 | -.017 | -.528^**^ | .093 |  |
| 27 | Cortisol change (log) | .081 | -.189 | -.099 | .011 | .223 | -.019 | .119 | .051 | -.061 | -.012 | -.066 | -.038 | .063 | .041 | -.211 | .081 | -.107 | .051 | -.038 | .138 | .055 | .046 | -.047 | .151 | -.314^**^ | .039 |
