# Supplementary material for: Football Fan Aggression: The Importance of Low Basal Cortisol and a Fair Referee
Source: PLoS One. 2015 Apr 6;10(4):e0120103. doi: 10.1371/journal.pone.0120103 (PMC4386810; doi:10.1371/journal.pone.0120103)
Supplement: S2 Table — (DOCX) [file pone.0120103.s002.docx]

| **Outcome variable** | **Rival fan video** | | | | | |
| --- | --- | --- | --- | --- | --- | --- |
|  | Positive | | Negative | | Neutral | |
|  | *M* | *SD* | *M* | *SD* | *M* | *SD* |
| Hot sauce administered (%) | 45.88 | 39.47 | 53.41 | 41.69 | 60.49 | 34.39 |
| Anger Change (post-pre) | 0.44 | 0.68 | 0.86 | 0.87 | 1.02 | 0.98 |
| Positive Mood Change (post-pre) | 0.03 | 0.45 | -0.15 | 0.40 | -0.06 | 0.55 |
| Negative Mood Change (post-pre) | 0.32 | 0.54 | 0.36 | 0.79 | 0.40 | 0.63 |
| Testosterone change (log) | -0.12 | 0.70 | -0.06 | 0.61 | -0.42 | 0.94 |
| Cortisol change (log) | -0.87 | 0.94 | -0.63 | 0.71 | -0.39 | 0.71 |
